# The dendritic SNARE fusion machinery involved in AMPARs insertion during long-term potentiation

**DOI:** 10.3389/fncel.2014.00407

**Published:** 2014-12-22

**Authors:** Sandra Jurado

**Affiliations:** Department of Pharmacology, University of Maryland School of MedicineBaltimore, MD, USA

**Keywords:** AMPARs, SNAREs, dendritic exocytosis, syntaxin-3

## Abstract

Sorting endosomes carry α-amino-3-hydroxy-5-methyl-4-isoxazolepropionic acid (AMPA)-type glutamate receptors (AMPARs) from their maturation sites to their final destination at the dendritic plasma membrane through both constitutive and regulated exocytosis. Insertion of functional AMPARs into the postsynaptic membrane is essential for maintaining fast excitatory synaptic transmission and plasticity. Despite this crucial role in neuronal function, the machinery mediating the fusion of AMPAR-containing endosomes in dendrites has been largely understudied in comparison to presynaptic vesicle exocytosis. Increasing evidence suggests that similarly to neurotransmitter release, AMPARs insertion relies on the formation of a SNARE complex (soluble NSF-attachment protein receptor), whose composition in dendrites has just begun to be elucidated. This review analyzes recent findings of the fusion machinery involved in regulated AMPARs insertion and discusses how dendritic exocytosis and AMPARs lateral diffusion may work together to support synaptic plasticity.

## Introduction

As integral membrane proteins, synaptic α-amino-3-hydroxy-5-methyl-4-isoxazolepropionic acid receptors (AMPARs) make use of the entire secretory pathway to reach their final destination at the postsynaptic density (PSD) of dendritic spines. In neurons, the endoplasmic reticulum (ER) can extend into dendrites where it serves as the site for protein biosynthesis as well as an internal calcium storage organelle (Torre and Steward, 1996; Spacek and Harris, 1997; Gardiol et al., 1999; Cui-Wang et al., 2012). These early trafficking steps through the secretory pathway greatly influence the number of available AMPARs since exit from the ER is a limiting step controlled by numerous signaling pathways (Standley et al., 2000; Scott et al., 2003; Hawkins et al., 2004; Horak et al., 2008). According to this notion, retention of AMPARs in the ER has been associated to impairments in synaptic potentiation elicited in CA3-CA1 synapses in the hippocampus (Broutman and Baudry, 2001). After departure from the ER, newly synthesized AMPARs reach the Golgi apparatus (GA) which in neurons is located both in the peri-nuclear region and in discrete Golgi outposts at dendritic branch points (Lowenstein et al., 1994; Horton and Ehlers, 2003; Horton et al., 2005; Ye et al., 2007). Following processing including glycosylation and peptide cleavage, mature AMPARs leave the GA in discrete membranous carriers, largely recycling endosomes (RE), which are then exocytosed at the dendritic plasma membrane. The fusion of these AMPAR-containing endosomes is believed to be highly regulated as it influences surface receptor composition and cell morphology. Two types of endosome exocytosis have been proposed: a constitutive recycling pathway that maintains an steady supply of lipids and membrane proteins and an activity-dependent fusion that underlies acute and long-term changes of molecular composition and synaptic function such as long-term synaptic potentiation (LTP) (reviewed in Shepherd and Huganir, 2007; Henley et al., 2011; Huganir and Nicoll, 2013).

**Figure 1:**
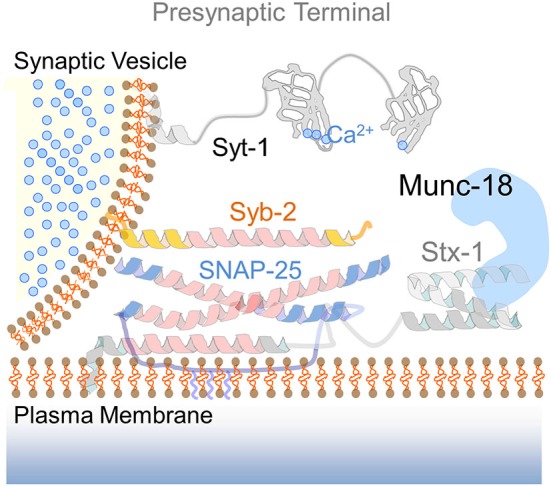
**The presynaptic SNARE complex**. Drawing of the SNARE fusion machinery mediating calcium-dependent exocytosis of synaptic vesicles. The cartoon illustrates the functional elements of the presynaptic SNARE complex: syntaxin-1 (Stx-1) in an open conformation via interaction with the SM protein Munc-18, SNAP-25 and synaptobrevin-2 (Syb-2). The calcium sensor synaptotagmin-1 (Syt-1) with two calcium-binding C2 domains is located at the vesicle membrane. Upon calcium entry, Syt-1 interacts with complexin (not shown) to promote neurotransmitter release.

The final step of intracellular membrane fusion is generally controlled by Sec1/Munc-18-like proteins (SM proteins) and the formation of a SNARE complex (Südhof, 2012). The assembly of the SNARE complex into a stable four-helix bundle occurs by the interaction of the SNARE motifs from syntaxin, synaptobrevin and SNAP proteins (Figure 1). SNARE complex formation is an exothermic process thought to provide the energy required for membrane fusion (Jahn and Scheller, 2006). According to their universal role in membrane fusion, previous work suggested that SNARE-dependent exocytosis mediates the fusion of AMPAR-containing endosomes with the postsynaptic membrane (Lledo et al., 1998; Lu et al., 2001; Kennedy et al., 2010; Jurado et al., 2013). However whereas the presynaptic SNARE fusion machinery has been identified, the composition of postsynaptic SNARE complexes has remained unclear until recently. Moreover, it is still uncertain whether the same pool of AMPARs-containing endosomes is capable of undergoing both constitutive and activity-dependent exocytosis via a similar SNARE fusion machinery. The identification of distinct SNARE molecules specifically involved in constitutive and/or regulated AMPARs insertion is particularly important since it may provide novel targets to selectively manipulate synaptic transmission and plasticity such as LTP which is thought to be implicated in learning and memory (Malenka and Bear, 2004; Neves et al., 2008). Recent efforts to elucidate the composition of postsynaptic SNAREs involved in activity-dependent exocytosis suggest that membrane fusion at the postsynaptic compartment is molecularly distinct from its presynaptic counterpart. Unfortunately, the fusion machinery underlying constitutive AMPARs insertion has received less attention despite its crucial role in maintaining basal synaptic strength. For this reason, here we primarily review data from experiments addressing the mechanism of AMPARs exocytosis during NMDAR-dependent LTP elicited in CA3-CA1 synapses in acute hippocampal slices or by activating N-methyl-D-aspartate (NMDA) receptors (NMDARs) in cultured neurons. NMDAR-dependent LTP is arguably the best studied form of long-term plasticity and whose deficit in different cell types and brain regions may contribute to several prominent neurological and neuropsychiatric disorders (Geschwind and Levitt, 2007; Kauer and Malenka, 2007; Clapp et al., 2012; Ehlers, 2012). In addition to discussing the fusion machinery of AMPARs-containing endosomes, we consider how regulated exocytosis may cooperate with other membrane processes such as receptors lateral diffusion to control the number of synaptic AMPARs, therein synaptic transmission and plasticity in the healthy brain.

## AMPARs exocytosis during LTP

In general, LTP can be elicited by brief repetitive stimulation of excitatory afferents (Bliss and Lomo, 1973; Malenka and Bear, 2004) which raises postsynaptic calcium levels mainly due to the activation of synaptic NMDARs (Collingridge et al., 1983; Kauer et al., 1988). Intensive research over the last three decades has demonstrated that postsynaptic calcium influx ultimately increases the number of synaptic AMPARs (Malenka and Bear, 2004; Huganir and Nicoll, 2013). However the role of calcium-dependent exocytosis during LTP has not been fully appreciated until more recently. Numerous signal transduction pathways were suggested to play a role in translating the calcium signal into LTP (Sanes and Lichtman, 1999; Malenka and Bear, 2004). Compelling evidence using genetic and pharmacological approaches indicated that calcium/calmodulin (CaM)-dependent protein kinase II (CaMKII) played a mandatory role in long-lasting increase of synaptic strength (Malenka et al., 1988; Malinow et al., 1989; Silva et al., 1992; Pettit et al., 1994; Lledo et al., 1995, 1998; Giese et al., 1998; Lisman et al., 2012). Due to the prominent role of CaMKII in LTP, it was initially assumed that AMPARs insertion was only indirectly regulated by calcium, in contrast to the calcium-regulated exocytosis observed in the presynaptic terminal. Electron microscopy studies revealed the presence of recycling endosomes in dendrites and dendritic spines (Cooney et al., 2002) suggesting that these dendritic vesicles may function as internal membrane stores of AMPARs. Furthermore, the presence of these dendritic endosomes occasionally observed as membrane-bound strongly suggested that they may interact and fuse with the plasma membrane to deliver their cargo in response to neuronal activity (Carroll et al., 1999; Lüscher et al., 1999; Beattie et al., 2000; Ehlers, 2000; Zhu et al., 2002; Park et al., 2004).

Early evidence that calcium-dependent synaptic potentiation requires a SNARE fusion machinery acting in dendrites came from LTP experiments in which botulinum neurotoxin B (BoNT/B), that cleaves synaptobrevins, and other inhibitor peptides of the SNARE complex were infused through the recording pipette (Lledo et al., 1998). Each of these inhibitors efficiently blocked the expression of LTP suggesting that exocytosis of AMPARs-containing endosomes is an essential step during synaptic potentiation. In parallalel to electrophysiological evidence for AMPARs exocytosis, the first optical demonstration of activity-triggered exocytosis in dendrites was reported (Maletic-Savatic and Malinow, 1998). In this pioneer study, neurons incubated with the lipophilic styryl dye FM1-43, a common reagent for the study of neurotransmitter release, incorporated the dye into postsynaptic compartments that destained within minutes upon neuronal stimulation (Maletic-Savatic and Malinow, 1998). These findings provided the first glimpse into postsynaptic exocytosis and suggested that dendritic vesicles may undergo activity-dependent fusion.

Over the years, advances on live cell imaging resulted in the appreciation of the morphological rearrangements that dendritic spines experience during synaptic potentiation. Structural plasticity of dendritic spines during LTP is often observed as a rapid increases of the spine head volume upon NMDAR activation, thus implying that membrane components are provided rapidly to support local growth (Murakoshi and Yasuda, 2012). Dendritic exocytosis of recycling endosomes containing surface receptors and other membrane proteins may provide an efficient way to support both synaptic and structural plasticity. According to this notion, live cell imaging studies using clostridial neurotoxins that disrupt SNARE complexes, or expression of dominant-negative SNARE proteins provided strong evidence for the role of activity-dependent exocytosis in supporting spine growth upon LTP induction (Park et al., 2004, 2006; Kopec et al., 2006, 2007; Yang et al., 2008).

Finally, recent work using shRNA-mediated knock-down of several SNARE proteins and high-resolution live cell imaging has confirmed the role of SNARE-dependent fusion during LTP (Kennedy et al., 2010; Jurado et al., 2013). Surprisingly despite the crucial role of CaMKII-dependent signaling in synaptic potentiation the exocytosis of AMPARs-containing endosomes may not be directly linked to CaMKII activity. Instead, postsynaptic exocytosis has been shown to require on small GTPases from the Ras and Rab families, which have been demonstrated to play a role in AMPAR mobilization upon NMDAR activation (Zhu et al., 2002). Numerous independent findings currently support the notion that AMPAR delivery to the plasma membrane is a calcium-regulated fusion event that involves activity-dependent exocytosis of AMPARs-containing endosomes (Lledo et al., 1998; Shi et al., 1999; Hayashi et al., 2000; Lu et al., 2001; Passafaro et al., 2001; Park et al., 2004; Makino and Malinow, 2009; Petrini et al., 2009; Kennedy et al., 2010; Ahmad et al., 2012; Jurado et al., 2013). Altogether, these data strongly support the hypothesis that regulated exocytosis of AMPAR-carrying vesicles may underlie both functional and structural aspects of synaptic potentiation.

## A postsynaptic SNARE complex for LTP

**Figure 2:**
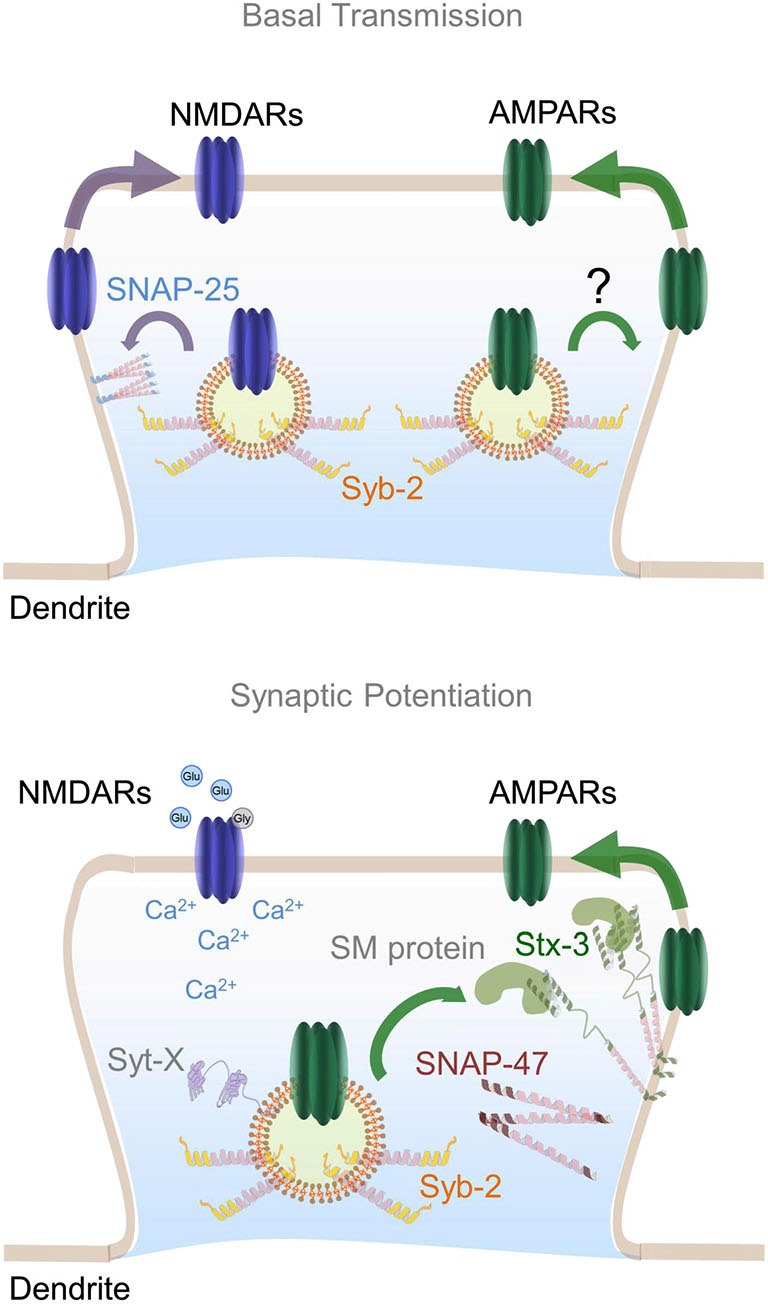
**Postsynaptic SNARE complex involved in AMPARs insertion during LTP**. Top panel represents dendritic SNARE proteins involved in constitutive trafficking of NMDARs and AMPARs. SNAP-25 is depicted as membrane-bound regulating constitutive NMDARs exocytosis whereas the vesicle SNARE synaptobrevin-2 (Syb-2) may be an integral component of both AMPARs and NMDARs-containing endosomes. Bottom panel illustrates the formation of a specific postsynaptic SNARE complex involved in AMPARs exocytosis upon NMDAR activation. SNAP-47 is shown in close proximity to syntaxin-3 which is anchored to the plasma membrane in an open conformation by its interaction with an unknown postsynaptic SM protein. In a similar fashion to SNAP-25, Syb-2 is depicted regulating constitutive recycling of AMPARs. Plasma membrane-bound Syntaxin-3 molecules may constitute micro-domains or hot spots for exocytosis of AMPARs-containing endosomes during LTP. Calcium influx into the postsynaptic terminal promotes the assembly of a SNARE complex constituted by Stx-3, SNAP-47 and Syb-2, as well as complexin (not shown) and a postsynaptic synaptotagmin isoform (Syt-X) still to be identified.

Membrane fusion events in eukaryotic cells are carried out by SNARE proteins. In neurons, the presynaptic SNARE complex is formed by the interaction of the vesicle SNARE protein (v-SNARE), synaptobrevin-2/vesicle-associated membrane protein 2 (Syb-2/VAMP2), and plasma membrane target SNARE proteins (t-SNAREs), syntaxin-1 and SNAP25 (Jahn and Fasshauer, 2012; Rizo and Südhof, 2012; Figure 1). Among the t-SNAREs, syntaxins exist in either a “closed” or an “open” conformation. Syntaxin “open” conformation must be achieved in order to form a functional SNARE complex. The conformational change of syntaxin-1 is facilitated by the interaction between its C-terminus and SM proteins (Khvotchev et al., 2007; Shen et al., 2007; Südhof and Rothman, 2009). In addition to this interaction, syntaxin-1 binds to small regulatory proteins known as complexins, this binding has been proposed to arrest the SNARE complex in a “primed” state until calcium influx invades the axon terminal and vesicles are finally fused (McMahon et al., 1995; Giraudo et al., 2006; Tang et al., 2006; Xue et al., 2008; Maximov et al., 2009). The final coupling of synaptic vesicle exocytosis to calcium is mediated by neuronal synaptotagmins, a family of transmembrane proteins with at least one calcium-binding domain (C2 domain) (Geppert et al., 1994; Fernández-Chacón et al., 2001; Pang et al., 2006; Xu et al., 2007). Calcium binding to synaptotagmin-1 C2 domains removes the complexin brake and promotes the binding of synaptotagmin to both the plasma membrane and the SNARE complex thereby triggering fusion (Rizo and Südhof, 1998; Tang et al., 2006; Südhof, 2012). Analogous to presynaptic fusion, AMPAR exocytosis has been shown to rely on SNAREs, although the composition of postsynaptic SNARE complexes involved in both constitutive and activity dependent recycling remains a topic of active research (Kennedy et al., 2010; Ahmad et al., 2012; Jurado et al., 2013) (please note the different molecular composition of the postsynaptic SNARE complex illustrated in Figure 2 to the canonical presynaptic SNARE complex in Figure 1). Consistent with this notion, complexins have emerged as important regulators of calcium-dependent exocytosis of AMPAR-carrying endosomes in LTP. Data from mice lacking complexin-2 provided early evidence to a potential role of complexins in LTP (Takahashi et al., 1999; Huang et al., 2000). More recently, the essential role of complexins in synaptic potentiation has been demonstrated using viral-mediated knock-down approaches *in vivo* (Ahmad et al., 2012). Ahmad et al. showed that complexins -1 and -2 control dendritic exocytosis of AMPARs during hippocampal LTP, although they may not be required for constitutive exocytosis. These findings further strengthen the involvement of a SNARE-dependent fusion during regulated AMPARs insertion.

## Composition of postsynaptic SNARE complex during LTP

In contrast to presynaptic neurotransmitter release, the composition of postsynaptic SNARE complexes mediating calcium-dependent AMPAR exocytosis in dendrites has just recently begun to be elucidated. In a similar fashion to axonal exocytosis, SNARE complexes in dendrites are constituted by three families of SNARE proteins: syntaxins, SNAPs and synaptobrevins. SNARE complex assembly is mediated by the interaction of the SNARE motifs, present in all SNARE proteins (note that two SNARE motifs are contributed by SNAP-25 and one by syntaxin-1 and synaptobrevin-2) (Hayashi et al., 1994; Sutton et al., 1998). Intriguingly, despite their specificity *in vivo* (Südhof and Rothman, 2009), SNARE-dependent interactions exhibit promiscuity *in vitro* (Fasshauer et al., 1999; Yang et al., 1999) suggesting that different SNARE complexes constituted by distinct combinations of syntaxin, SNAP and synaptobrevin isoforms may coexist within the same cell to regulate fusion events at different subcellular compartments. Moreover, different SNARE isoforms of for example membrane-bound syntaxins may sort distinct SNARE complexes to discrete membrane compartments or regions within the plasma membrane (i.e., postsynaptic membrane vs. presynaptic membrane) indicating hot spots for exocytosis. Consisting with this, immunohistochemistry and electron microscopy studies have exposed the presence of several SNAREs proteins at the somato-dendritic region in different brain areas. For example, syntaxin-3, SNAP-25 and synaptobrevin-2 have been detected in dendrites of nigral dopaminergic neurons which is indirect evidence for their involvement in dendritic secretion of neuromodulators like dopamine or perhaps neuropeptides (Witkovsky et al., 2009). Additionally, distinct SNARE-dependent mechanisms seem to underlie calcium-dependent fusion of secretory vesicles from axons and dendrites in hypothalamic neurons (Landry et al., 2003). Below, we review our current knowledge of SNAREs involved in AMPARs insertion during LTP following a chronological order from the first evidence for the role of postsynaptic synaptobrevins to the more recent work in SNAP proteins and syntaxins.

### Postsynaptic synaptobrevins

Synaptobrevin -1 and -2 isoforms are small transmembrane proteins that belong to a larger VAMP family (Ernst and Brunger, 2003; Brunger et al., 2009). Interestingly, synaptobrevin-2 has long been known to undergo transcytosis, an early intracellular trafficking event that temporally drives axonal vesicles to the dendritic compartment (Sampo et al., 2003; Wisco et al., 2003; Yap et al., 2008; Ascaño et al., 2009). Identification of vesicles containing classical presynaptic molecules in dendrites, even if only temporarily during early development, raised the intriguing possibility that proteins critical for presynaptic function may also act at postsynaptic locations. According to this idea, some of the first evidence that AMPARs insertion during LTP requires postsynaptic exocytosis came from experiments where synaptobrevin-mediated fusion was disrupted using botulinum toxin B which cleaves VAMP family SNARE proteins infused into postsynaptic neurons via the recording pipette (Lledo et al., 1998). This early observation led to a model where AMPAR-containing endosomes fuse with the plasma membrane upon LTP induction (Figure 2).

In addition to functional plasticity, several studies have shown that postsynaptic exocytosis likely mediated by postsynaptic synaptobrevins is required for structural plasticity at glutamatergic synapses (Park et al., 2004, 2006; Kopec et al., 2006, 2007; Yang et al., 2008). Upon NMDARs activation dendritic spines have been shown to increase their volume (Murakoshi and Yasuda, 2012). This stimulus-induced spine growth is blocked by the infusion of botulinum toxin B or expression of dominant-negative SNARE proteins in postsynaptic neurons (Park et al., 2006; Kopec et al., 2007; Yang et al., 2008), indicating that SNARE complex-mediated membrane fusion is required for both structural and synaptic plasticity.

More recently, experiments in cultures prepared from synaptobrevin-2 KO mice indicated that synaptobrevin-2 contributes to maintaining both synaptic and extrasynaptic AMPARs (Jurado et al., 2013). This observation raises the question of which SNARE proteins control the constitutive and regulated delivery of AMPARs to the plasma membrane. Synaptobrevin-2 may be a component of the AMPAR-containing organelles involved in both pathways, although other R-SNAREs must contribute as well since surface levels of AMPARs were only partly reduced in cells lacking synaptobrevin-2.

### Postsynaptic SNAPs

#### SNAP-25

A functional SNARE-complex requires at least one copy of the plasma membrane-associated SNAREs known as SNAPs (Synaptosomal-associated proteins), with SNAP-25 being the canonical protein at the presynapse (Jahn and Scheller, 2006; Rizo and Rosenmund, 2008; Südhof and Rothman, 2009). Immunohistochemistry in cultured hippocampal neurons have identified several SNAP isoforms in dendrites, including SNAP-25 in similar fashion to other SNAREs which exhibit ubiquitous expression patterns (Südhof, 2012). Moreover, SNAP-25 has even been found in PSD fractionations suggesting a role in dendritic membrane fusion (Jordan et al., 2004; Chen et al., 2006). According to this, *in vivo* knock-down of SNAP-25 impairs NMDAR-mediated transmission in slices and decreases synaptic NMDAR levels in cultured neurons without affecting basal transmission or AMPARs levels (Jurado et al., 2013). These findings are consistent with previous work that shows a role of SNAP-25 in NMDAR trafficking (Lau et al., 2010). Taken together, these results indicate a rather specific role of SNAP-25 in regulating NMDAR-containing endosomes and therefore in controlling the threshold of NMDAR-dependent LTP induction. Furthermore, these findings support the hypothesis that NMDARs and AMPARs are transported via distinct vesicles (Fong et al., 2002; Washbourne et al., 2002) and are sorted via different intracellular pathways to synaptic sites (Jeyifous et al., 2009). Whereas AMPARs are believed to undergo forward trafficking to the plasma membrane via the GA likely through dendritic Golgi outpost, NMDAR may traffic via nonconventional secretory pathway involving CASK and SAP97 (Jeyifous et al., 2009).

#### SNAP-23

A role for SNAP-23, a SNAP-25 homolog, in glutamate receptor trafficking has been recently suggested. Using immunohistochemistry, Suh et al., showed that endogenous SNAP-23 is highly enriched in dendrites and dendritic spines (Suh et al., 2010). Furthermore, postsynaptic knock-down of SNAP-23, but not SNAP-25, reduced the size of NMDA-evoked currents without affecting presynaptic glutamate release in cultured hippocampal slices. These findings suggest that SNAP-23 may influence AMPARs exocytosis indirectly by regulating surface NMDARs and thereby modulating the induction of synaptic potentiation. However a SNAP-23 shRNA introduced *in vivo* did not subsequently affect LTP in acute hippocampal slices (Jurado et al., 2013). This apparent contradiction may be explained by the use of robust induction protocols to elicit LTP in acute slices in comparison to the milder protocols required to induce potentiation in cultured neurons. Nevertheless, neither SNAP-23 nor SNAP-25 seem to play a direct role in regulated exocytosis of AMPAR-containing endosomes during LTP, although may affect plasticity by controlling NMDAR function.

#### SNAP-47

SNAP-47, a newly identified SNAP protein (Holt et al., 2006), has been showed to play a role in LTP using an *in vivo* knock-down strategy (Jurado et al., 2013). Immunocytochemistry and structured illumination microscopy have revealed a widespread distribution of endogenous SNAP-47 in both neuronal cell bodies and neuronal processes (Holt et al., 2006; Jurado et al., 2013). Importantly, SNAP-47 knock-down did not alter basal AMPAR- or NMDAR-mediated synaptic responses or basal AMPAR surface expression, providing evidence for a specific role of SNAP-47 in activity-dependent AMPAR exocytosis but not in constitutive trafficking. Moreover, as a genuine SNARE, SNAP-47 has been shown to assemble into stable SNARE complexes with syntaxin-1 and synaptobrevin-2 *in vitro* (Holt et al., 2006). According to this, mutagenesis of SNAP-47 confirmed that a SNARE-dependent interaction is critical for its role in LTP (Jurado et al., 2013).

Sequence comparison of SNAP-47 with other SNAP-25 homologs has revealed SNAP-47 unusual structure that may reflect its functional specialization at the postsynaptic site. SNAP-47 has a long N-terminal stretch and an extended loop between its two SNARE motifs. Also, in contrast to SNAP-23 and SNAP-25, which are predominantly bound to the plasma membrane, SNAP-47 lacks an immediately identifiable membrane anchor sequence which suggest it may be partly cytosolic (Holt et al., 2006). These structural differences of SNAP-47 may be advantageous for regulating membrane fusion at subcellular locations where exocytotic domains are not permanent but rather transiently defined (Yudowski et al., 2007; Yang et al., 2008; Petrini et al., 2009; Patterson et al., 2010).

### Postsynaptic syntaxins

More recent efforts to elucidate the identity of the postsynaptic SNARE complex have been dedicated to the characterization of postsynaptic syntaxins. Syntaxins are small transmembrane proteins that comprised a family of 15 members from which only four (syntaxin 1–4), localize to the plasma membrane where they cluster into microdomains that may support SNARE complex assembly (Lang et al., 2001; Ohara-Imaizumi et al., 2004; Low et al., 2006; Sieber et al., 2006, 2007; Kennedy et al., 2010). These features suggest that identification of syntaxin clusters in dendrites may provide clues to the exact location of AMPARs exocytosis.

Given the prominent role of complexins in calcium-dependent dendritic fusion (Takahashi et al., 1999; Huang et al., 2000; Ahmad et al., 2012), it is reasonable to assume that a syntaxin capable of interacting with complexin (Pabst et al., 2000) will be implicated in LTP. Consistent with this logic, syntaxin-3 has been recently proposed to control AMPARs insertion via a complexin-dependent mechanism (Jurado et al., 2013). Analysis of LTP elicited in acute hippocampal slices from mice expressing shRNAs against different syntaxins revealed that syntaxin-3, but not -1 or -4, plays a critical role in LTP but does not participate in constitutive or presynaptic exocytosis. Structured illumination microscopy showed a relatively ubiquitous distribution of endogenous syntaxin-3 including dendrites and cell bodies. Interestingly, the same syntaxin-3 shRNA in dissociated hippocampal neurons blocked the increase in surface expression of endogenous AMPARs upon NMDAR activation, a cell culture model of LTP (Lu et al., 2001; Passafaro et al., 2001; Park et al., 2004). More importantly, both the block of LTP and AMPARs insertion were rescued by reintroducing syntaxin-3 which rules out potential off-target effects of the shRNA being used. Further structure/function analysis replacing endogenous syntaxin-3 by a non complexin-binding mutant confirmed that syntaxin-3/complexin interaction is necessary for the function of postsynaptic SNARE complexes implicated in AMPARs exocytosis. These results suggest that postsynaptic syntaxin-3 via complexins may constitutively restrict AMPARs insertion until calcium influx reaches the postsynaptic compartment in a similar fashion to their function at presynaptic terminals (Giraudo et al., 2006; Tang et al., 2006; Huntwork and Littleton, 2007; Maximov et al., 2009; Xue et al., 2009; Yang et al., 2010). Furthermore, in a manner analogous to syntaxin-1 in presynaptic terminals, syntaxin-3 was shown to require the binding of SM proteins. This requirement of postsynaptic SM proteins was shown using a molecular replacement strategy in which a syntaxin-3 mutant with a deletion of the SM-binding sequence was ineffective to restore synaptic potentiation in the absence of endogenous syntaxin-3 (Jurado et al., 2013). These findings suggest that a postsynaptic Munc18-like protein still to be identified is likely to catalyze the assembly of the postsynaptic SNARE complex involved in LTP.

Surprisingly syntaxin-4 a syntaxin isoform that does not bind to complexin (Pabst et al., 2000), has also been suggested to mediate AMPARs exocytosis (Kennedy et al., 2010). This evidence is primarily supported by the block of recycling endosomes exocytosis marked with superecliptic pHluorin (SEP)-fused transferrin receptors (TfR-SEP) by a specific syntaxin-4 shRNA. This apparent discrepancy may in large part be explained by the differences in the methods used to assay the fusion of AMPARs-containing endosomes, as endogenous AMPARs like those assayed by electrophysiology may traffic differently from overexpressed recombinant receptors. Nonetheless, these results raise the intriguing possibility that different syntaxin isoforms may coexist in postsynaptic compartments and sort different cargos via independent microdomains (Puthenveedu et al., 2010; Temkin et al., 2011).

## Location and timing of AMPARs exocytosis

Although the role for postsynaptic exocytosis in synaptic plasticity is now clear, the specific locations and timing of AMPARs exocytosis continue to be an active matter of debate. Most studies exploring this issue have yielded inconsistent results (Gerges et al., 2006; Kopec et al., 2006, 2007; Park et al., 2006; Yudowski et al., 2007; Yang et al., 2008; Lin et al., 2009; Makino and Malinow, 2009; Petrini et al., 2009; Kennedy et al., 2010; Opazo et al., 2010; Patterson et al., 2010; Tanaka and Hirano, 2012). While some have suggested that activity stimulates exocytosis in the soma and dendritic shafts (Yudowski et al., 2007; Yang et al., 2008; Lin et al., 2009; Makino and Malinow, 2009; Petrini et al., 2009; Opazo et al., 2010; Opazo and Choquet, 2011; Tanaka and Hirano, 2012), others support insertion directly into stimulated dendritic spines (Gerges et al., 2006; Kopec et al., 2006; Park et al., 2006; Kennedy et al., 2010; Patterson et al., 2010).

Early work to determine the timing of AMPARs exocytosis used an irreversible photoactivable AMPAR inhibitor to analyze the exchange rate of synaptic or extrasynaptic AMPARs upon electrical stimulation or glutamate uncaging (Adesnik et al., 2005). Surprisingly, exchange of synaptic AMPARS took place only after several hours, a timescale much slower than previously thought. In contrast, AMPAR currents measured at the cell body by glutamate uncaging recovered within minutes, suggesting more rapid cycling of receptors at the neuronal soma under basal conditions (Adesnik et al., 2005). Unfortunately, no direct measurements of endogenous AMPAR exocytosis exist, and its time course in living synapses remains unknown. Although electrophysiology experiments are useful to assay the timing and functional relevance of dendritic exocytosis, determining the location of AMPARs insertion requires imaging technologies. Efforts to visualize the location of AMPARs exocytosis have largely relied on optical probes based on SEP, a pH-sensitive GFP variant, which is fluorescent at neutral pH but is quenched when inside acidic vesicles (Miesenböck et al., 1998). SEP-labeled AMPARs, particularly GluA1 subunit-containing receptors, have been used in a number of studies to directly identify AMPARs exocytosis in dendrites (Kopec et al., 2006, 2007; Yudowski et al., 2007; Jaskolski et al., 2009; Lin et al., 2009; Makino and Malinow, 2009; Araki et al., 2010; Kennedy et al., 2010; Patterson et al., 2010). Two-photon glutamate uncaging at individual dendritic spines has revealed that SEP-GluA1 is inserted in the dendritic shaft in neighboring areas of activated spines (Makino and Malinow, 2009). Conversely, a recent study demonstrated that exocytosis of AMPARs-containing endosomes occurs within spines (Kennedy et al., 2010). This last study used transferrin, a marker for recycling endosomes, to demonstrate that endosomes already present in dendritic spines undergo fusion similarly to those in the dendritic shaft. Differences in experimental and imaging conditions most likely underlie the disparity of results obtained using these visualization approaches. Nonetheless, these collective data have been incorporated into a prominent hypothesis in the field that postulates that AMPARs are first inserted into the extra/peri-synaptic surface, then diffuse laterally to the PSD (Borgdorff and Choquet, 2002; Ehlers et al., 2007; Yudowski et al., 2007; Heine et al., 2008; Makino and Malinow, 2009), where they are retained by interactions with scaffold proteins (Henley et al., 2011; MacGillavry et al., 2011; Opazo and Choquet, 2011). In this scenario AMPARs exocytosis is required to replenish the peri-synaptic pool of freely moving surface receptors that will be sequestered by PSD scaffolds during potentiation.

Related to the issue of the location of AMPARs insertion is the question whether AMPARs exocytosis is required for LTP induction or just for LTP maintenance. First experiments using postsynaptic loading of SNARE inhibitors showed that membrane fusion inhibition was effective in shortening the duration of LTP without affecting induction (Lledo et al., 1998). These results support the notion that exocytosis may be critical for LTP maintenance by supplying the pool of surface AMPARs that can then freely diffuse to synaptic locations. However, recent evidence from *in vivo* molecular manipulations of several SNARE proteins and complexins has shown an almost complete block of LTP right after stimulation (Ahmad et al., 2012; Jurado et al., 2013) suggesting that early insertion of AMPARs may be necessary for eliciting synaptic plasticity. A potential explanation for this apparent conflict may be the different methods used for blocking exocytosis. Detection of synaptic effects using acute infusions in the cell body may be delayed by the necessity of the infused molecule to reach the specific synapses that are being stimulated. Future work in this topic is guaranteed which will provide answers to these questions likely by employing novel cutting-edge visualization techniques such as super-resolution microscopy.

## Concluding remarks

Despite the fact that SNARE-dependent fusion machinery is involved in both pre and postsynaptic exocytosis, there are important differences in the properties of fast neurotransmitter release and activity-dependent AMPARs insertion during LTP. In presynaptic terminals, small synaptic vesicles are docked at the plasma membrane in specialized active zones and primed such that fusion occurs rapidly, within milliseconds following a rise in calcium. In the other side of the synapse, AMPARs-containing endosomes are not tightly coupled to the dendritic plasma membrane but instead may require myosin-dependent trafficking into dendritic spines (Correia et al., 2008; Wang et al., 2008) which would explain the slow exocytosis kinetics in the range of seconds or minutes (Yudowski et al., 2007; Yang et al., 2008; Petrini et al., 2009; Patterson et al., 2010). The reported differences in the composition of the postsynaptic SNARE complex vs. its presynaptic counterpart could account for these significant functional differences. Moreover, all known membrane fusion reactions that require complexin also require a synaptotagmin isoform (Xu et al., 2007; Cai et al., 2008; Schonn et al., 2008) which suggests that a postsynaptic synaptotagmin may control calcium-dependent synaptic plasticity. Interestingly, synaptotagmin-1, the major trigger of fast neurotransmitter release, is not required for LTP (Ahmad et al., 2012) implying that a different synaptotagmin still to be identified could be involved.

In summary, multiple SNAREs have been found in dendrites where they seem to play an essential role in controlling the constitutive and regulated exocytosis of glutamate receptors. Particularly, we have reviewed convincing evidence suggesting that the t-SNARE proteins Stx-3 and SNAP-47 and the v-SNARE protein synaptobrevin-2 are essential components of the postsynaptic vesicle fusion machinery that is required for LTP. Furthermore, postsynaptic synaptobrevin-2 may also contribute to constitutive postsynaptic AMPAR trafficking, and a postsynaptic SNARE complex constituted by SNAP-25 and/or SNAP-23 may control constitutive trafficking of NMDARs (Figure 2). Future efforts to elucidate the detailed molecular mechanisms including postsynaptic synaptotagmins and SM proteins involved in both synaptic transmission and plasticity will be critical for understanding the neural basis of many aspects of normal and pathological brain function.

## Conflict of interest statement

The author declares that the research was conducted in the absence of any commercial or financial relationships that could be construed as a potential conflict of interest.
